# Genomic Frontiers in Alzheimer's Research: A Primer on the Alzheimer's Disease Sequencing Project and its AI/ML Opportunities

**DOI:** 10.1002/alz70855_101956

**Published:** 2025-12-23

**Authors:** Emily Greenfest‐Allen, Yuk Yee Leung, Wan‐Ping Lee, Amanda Kuzma, Otto Valladares, William S Bush, Eden R. Martin, Adam C. Naj, Gerald D. Schellenberg, Li‐San Wang

**Affiliations:** ^1^ Penn Neurodegeneration Genomics Center, Department of Pathology and Laboratory Medicine, Perelman School of Medicine, University of Pennsylvania, Philadelphia, PA, USA; ^2^ Department of Population and Quantitative Health Sciences, Institute for Computational Biology, Case Western Reserve University, Cleveland, OH, USA; ^3^ Dr. John T. Macdonald Foundation Department of Human Genetics, University of Miami Miller School of Medicine, Miami, FL, USA; ^4^ University of Pennsylvania, Perelman School of Medicine, Philadelphia, PA, USA; ^5^ Penn Neurodegeneration Genomics Center, University of Pennsylvania, Perelman School of Medicine, Philadelphia, PA, USA; ^6^ Penn Neurodegeneration Genomics Center, Department of Pathology and Laboratory Medicine, University of Pennsylvania Perelman School of Medicine, Philadelphia, PA, USA

## Abstract

**Background:**

In this introductory talk, we embark on a journey through the genomic frontiers of Alzheimer's disease (AD) research, illuminating the wide range of opportunities the pioneering Alzheimer's Disease Sequencing Project (ADSP) offers to AI/ML researchers. From predictive modeling to identifying novel biomarkers and therapeutic targets, we will explore the vast landscape of possibilities for ADSP data‐driven discoveries that can help shape the future of Alzheimer's research and precision medicine.

**Method:**

ADSP integrates numerous projects that collectively unravel the intricate genetic landscape of AD with the primary objective of advancing precision medicine for the millions affected globally by this devastating disease. Working toward the goal of sequencing and analyzing up to 150,000 complete genomes and associated clinical and functional data in the next five years, ADSP has amassed an unprecedented wealth of genomic data from diverse populations, providing a comprehensive and holistic understanding of the genetic underpinnings of AD.

**Result:**

This presentation serves as a primer, exploring various components of the ADSP and discussing the unprecedented resources it presents AI/ML researchers: (1) Diversity Initiative: The ADSP places a paramount emphasis on diversity, ensuring the inclusion of a wide range of populations in its genomic dataset. The current (release 5) dataset includes whole genomes from 57,302 unique participants, including 6,875 with African ancestry, 5,523 with Asian, and 15,390 Hispanic/Latino individuals. (2) Phenotype Harmonization: Harmonizing phenotypic data across diverse cohorts is a critical aspect of the ADSP, facilitating meaningful comparisons and analyses. (3) Open Access Initiative: unrestricted access to harmonized summary statistics, curated genetic associations, and functional annotations enables generation of task‐ and domain‐specific knowledge bases. (4) Functional Genomics: Moving beyond genetic variations, the ADSP incorporates functional genomics to discern the biological mechanisms at play.

**Conclusion:**

The potential of AI/ML within the ADSP serves to emphasize the need for collaborative initiatives between AI/ML researchers and the broader Alzheimer's research community. The synergy between these fields holds the key to unlocking breakthroughs that can translate genomic insights into tangible clinical advancements.

